# Identification of potential immunotherapeutic targets in antigen presentation and costimulation networks

**DOI:** 10.1186/2051-1426-2-S3-P126

**Published:** 2014-11-06

**Authors:** Robbert Spaapen, Vincent Blomen, Peter van Veelen, Marlieke Jongsma, Lennert Janssen, George Janssen, Matthijs Raaben, Thijn Brummelkamp, Jacques Neefjes

**Affiliations:** 1 Sanquin Research, Netherlands; 2 The Netherlands Cancer Institute, Netherlands; 3 Leiden University Medical Center, Netherlands

## 

Anti-tumor immune responses are often hampered by an excess of coinhibition of T cells, but also by a lack of MHC antigen presentation and costimulation. The latter are not yet successfully exploited as targets for immunotherapy, while augmenting MHC and costimulatory molecules may favorably direct adaptive immune responses against cancer. Thus we set off to identify novel molecular targets within the network of antigen presentation and costimulation.

As our understanding of players of MHC class I (MHC-I) is far from complete, we initially performed an unbiased genome-wide screen to systematically decipher the rest of the MHC-I network. The screen is based on near-haploid human leukemic cells (KBM7), retrovirally modified to contain millions of different single-gene-knockout cells. As a result some of these cells became MHC-I high or low and were separately sorted out by FACS. Deep sequence analyses of the sorted cell populations versus unsorted control cells revealed that the MHC-I low population was enriched for knockouts of known key proteins in the MHC-I network, such as Tapasin, TAP1, TAP2 and β2M. Among the new hit proteins of both screens, we identified two enzymes: a novel peptidase and an E3 ligase, which were first validated using siRNA knockdown in different cell lines.

Using cutting edge TALEN and CRISPR genome-editing technology, we generated different cell lines that are completely knockout for the peptidase and E3 ligase. As expected, MHC-I expression on the E3 ligase knockout cell line was increased. Expression on the peptidase knockout cell lines was drastically decreased and could be rescued by retroviral reconstitution of the peptidase. Importantly, reconstitution using a catalytic inactive peptidase did not rescue MHC-I expression, showing that the proteolytic activity is essential for its MHC-I related function.

Treatment of peptidase knockout cells with IFN-γ revealed that MHC-I could still be up-regulated. Moreover, the peptidase itself was not IFN-γ inducible in contrast to known other MHC-I regulators, arguing for an unexpected immune-independent regulation of the antigen presentation pathway.

We are currently in the process of performing similar screens for the cell surface expression of costimulatory molecules. In conclusion, using high-tech screening and the latest state-of-the-art genome-editing tools, we are building a near-complete map of the intracellular network behind MHC-I and costimulatory molecule cell surface expression. Thereby we have initiated new efforts to define novel potential targets for immunotherapy.

